# Cell Microenvironment Engineering and Monitoring for Tissue Engineering and Regenerative Medicine: The Recent Advances

**DOI:** 10.1155/2014/921905

**Published:** 2014-07-20

**Authors:** Julien Barthes, Hayriye Özçelik, Mathilde Hindié, Albana Ndreu-Halili, Anwarul Hasan, Nihal Engin Vrana

**Affiliations:** ^1^Institut National de la Santé et de la Recherche Médicale (INSERM) UMR-S 1121, “Biomatériaux et Bioingénierie”, 11 rue Humann, 67085 Strasbourg Cedex, France; ^2^Equipe de Recherche sur les Relations Matrice Extracellulaire-Cellules, Université de Cergy-Pontoise, 2 Avenue Adolphe Chauvin, 95302 Cergy Pontoise, France; ^3^Department of Computer Engineering, Epoka University, Tirana, Albania; ^4^Biomedical Engineering and Department of Mechanical Engineering, American University of Beirut, Beirut 1107 2020, Lebanon; ^5^Center for Biomedical Engineering, Department of Medicine, Brigham and Women's Hospital, Harvard Medical School, Cambridge, MA 02139, USA; ^6^Harvard-MIT Division of Health Sciences and Technology, Massachusetts Institute of Technology, Cambridge, MA 02139, USA; ^7^Protip SAS, 8 Place de l'Hôpital, 67000, Strasbourg, France

## Abstract

In tissue engineering and regenerative medicine, the conditions in the immediate vicinity of the cells have a direct effect on cells' behaviour and subsequently on clinical outcomes. Physical, chemical, and biological control of cell microenvironment are of crucial importance for the ability to direct and control cell behaviour in 3-dimensional tissue engineering scaffolds spatially and temporally. In this review, we will focus on the different aspects of cell microenvironment such as surface micro-, nanotopography, extracellular matrix composition and distribution, controlled release of soluble factors, and mechanical stress/strain conditions and how these aspects and their interactions can be used to achieve a higher degree of control over cellular activities. The effect of these parameters on the cellular behaviour within tissue engineering context is discussed and how these parameters are used to develop engineered tissues is elaborated. Also, recent techniques developed for the monitoring of the cell microenvironment *in vitro* and *in vivo* are reviewed, together with recent tissue engineering applications where the control of cell microenvironment has been exploited. Cell microenvironment engineering and monitoring are crucial parts of tissue engineering efforts and systems which utilize different components of the cell microenvironment simultaneously can provide more functional engineered tissues in the near future.

## 1. What is Cell Microenvironment?

Tissue engineering and regenerative medicine fields aim to produce artificial tissues or whole organs for both clinical applications and drug testing, disease models, and cell based biosensors. Even though there are several methods to approach tissue engineering, whether scaffold/biomaterial based approaches, utilization of decellularized natural materials, or scaffold-free methods, presence of the cellular component is inevitable [1]. As the advances in the different fields of biology demonstrated well that cells are highly sensitive to their environment, it can be said that the control over cell microenvironment is a fundamental aspect of tissue engineering and regenerative medicine.

Cell microenvironment is constituted by factors that directly affect conditions around a cell or group of cells which have direct or indirect effect on cell behavior via biophysical, biochemical, or other routes. When considered for a single cell* in vivo*, cell microenvironment is composed of (i) extracellular matrix (ECM), (ii) homotypic or heterotypic cells surrounding the single cell, (iii) cytokines, hormones, and other bioactive agents around the cells due to autocrine, endocrine, and paracrine secretions, (iv) nano/microscale topography and physical properties of the adjuvant cells and the ECM, and (v) mechanical forces caused by the movement of the organism or the movement of the physiological fluids such as blood. All these have a compound effect on the behavior of the cells, where the relative importance of each component is tissue and cell type dependent, and the next generation of engineered tissues must imitate these effects as much as possible to be functional in their target areas as a long term clinical solution.

**Figure 1:**
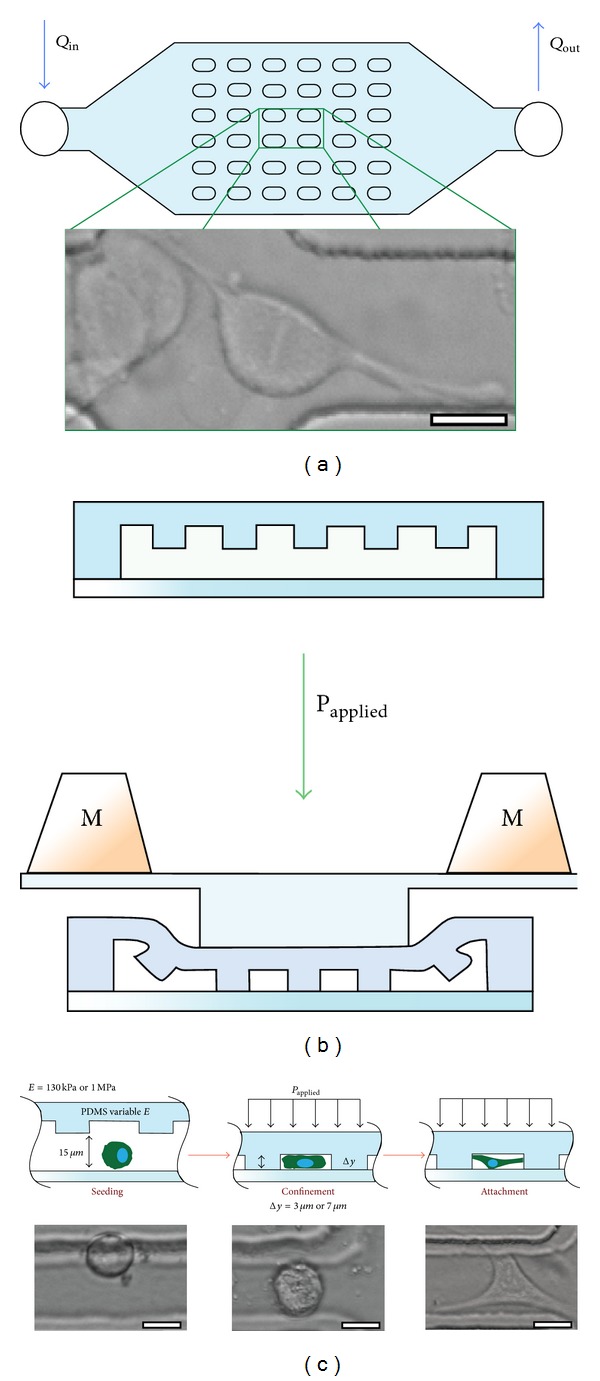
The effect of microlevel mechanical confinement on the division of HeLa cells. (a) and (b) show the macroscopic structure of the microfluidic system and the cross-section of PDMS posts. By the application of pressure on the posts, cells can be confined within the area between the posts (the distance between the posts is 40 *μ*m) (c). The confinement caused significant changes in the behavior of the cells during mitosis, such as delays in mitosis, and led to daughter cells of different sizes and multidaughter cells following mitosis. Reproduced from [4].

Cell microenvironment has many aspects and their control can result in substantial changes in cell behavior. For example, Satyam et al. showed that by macromolecular crowding in the cell microenvironment the secretion of ECM molecules can be significantly improved for corneal fibroblasts [2]. Another recent work shows that nanoscale, micropatterned, and highly flexible membranes can be used to develop retinal pigment epithelium layers for minimally invasive implantation within the eye [3]. By mechanically confining cells in a microfluidic platform, researchers were also able to control the mitotic processes (Figure 1) [4].

Microenvironment of stem cells is a particularly important topic in tissue engineering and regenerative medicine, as they are currently the most technically feasible source which can provide the large amount of cells needed for engineering clinically relevant amounts of tissue. Stem cell reservoirs are available for replenishment of the tissue in tissues in human body. The microenvironmental control over how these cells can keep their plasticity [5], that is, how they can stay quiescent and be utilized by the body only in case of necessity under healthy conditions, is a benchmark that needs to be met by engineered tissues. Moreover, failure to control the microenvironment of stem cells can also have deleterious effects such as dedifferentiation and subsequent tumor growth.

Another important concept related to the mimicking of tissue microenvironment is multidimensionality as most of the components of tissues have multidimensional order and orientation, which necessitates mimicry to achieve their function [6]. Multidimensionality is also an important aspect of other uses of tissue engineering, namely, model tissues and organs for pharmaceutical testing and also fundamental research. These microorgan structures should match the dimensional properties of the tissue and the organ they represent [7].

In this review, we will focus on different aspects of cell microenvironment and their direct effects on tissue engineering applications with particular focus on osteogenesis and angiogenesis. Each component of the cell microenvironment will be discussed separately and also in conjunction with the other components.

## 2. Micro/Nanopatterning for Microenvironment Engineering

All the cells in the human body are surrounded by topographical and biochemical signals. The physical structures comprise nanopores, nanofibers, and nanocrystals. Some examples of such structures in physiological settings are nanopores in capillaries, nanofibers in the basement membrane, and nanocrystals in the form of hydroxyapatite in the bone microstructure. Aligned cells are very prevalent in the tissue. For instance, maintenance of cell alignment is essential for muscle, cardiovascular/blood vessel, and corneal and nerve tissue engineering in which the controlling tissue microarchitecture and biological function are tightly connected. Various strategies have been developed to induce cell alignment, including topographical patterning (e.g., micro- and nanogrooves and aligned nanofibers), chemical treatment (patterns with cell-adhesive or repellent chemistries), controlled stress/strain conditions (e.g., stretching, fluid shear stress, and compression), and a combination of these methods.

From topography point of view, recent advances in micro- and nanofabrication enabled development of complex surface features by controlling their pattern, periodicity, shape, and dimensional properties. Today, design and construction of substrates with well-controlled physical and chemical properties and micro- and nanoarchitecture have become an important tool in the construction of tissue engineered replacements. Several top-down and bottom-up techniques such as phase separation, self-assembly, thin film deposition, chemical vapor deposition, chemical etching, nanoimprinting, photolithography, scanning probe lithography, and electron beam lithography [11] can be used in order to tailor micro- and nanoscale structured environments (scaffolds/surfaces) to stimulate cell growth and guide tissue regeneration in much the same way the extracellular matrix (ECM) does.

It is well known that cells can align along micro- and nanosized parallel grooves/ridges patterns [12–19]. Several studies indicate that alignment occurs when the periodicity and dimensions of the patterns are above a critical value. For example, Loesberg et al. [20] have shown that the fibroblasts did not show noticeable alignment with groove depths around 35 nm and ridges narrower than 100 nm. In another study, 100 nm depth was determined as a threshold for alignment of cardiomyocytes [21], osteoblast-like cells [16, 22], and hepatoblastoma cells [23]. On microgrooved surfaces, groove depth is one of the most important parameters in defining cell alignment. The degree of alignment of the cells along the microscale grooves is generally proportional to groove depth and inversely proportional to groove/ridge width if the other parameters are fixed [24]. On the other hand, Glawe et al. [25] studied that effect of high aspect ratio (aspect ratio = groove depth/groove width) microchannels with varying widths (20–60 *μ*m) on the alignment of smooth muscle cells. It was observed that alignment was dependent on the channel width, and narrow microchannels (20 *μ*m and 30 *μ*m) promote alignment of smooth muscle cells. On nanogrooved substrates, cell orientation was also found to be also less sensitive to groove width (90 to 500 nm) with MG-63 cells and C3A cells [16, 22]. When the ridges are smaller than that of focal adhesions (0.25–0.5 *μ*m wide and 2.0–10.0 *μ*m long), cell alignment is inhibited. Nanogrooves were too narrow for the cells to descend into the bottom of grooves. Thus, the focal adhesions and actin filaments are localized on the ridges. However, for vascular smooth muscle cells, channel widths as small as 332.5 nm have been shown to induce alignment and subsequent mechanical property improvement in the direction of alignment [26].

The lack of data on how height and groove width or quantitative interaction of these parameters which determine the degree of cell orientation have forced researchers to establish aspect ratio dependent models. For example, Kemkemer et al. [27] developed a model for predicting the cell orientation for cases where the cell is larger than the grooves. According to this, the square of the product of groove depth and spatial frequency or the aspect ratio for symmetric grooves were found to be the important features for alignment. In another study, Crouch et al.  [28] proposed a simple model to explain the relationship between aspect ratio and cell behavior on gratings with varying widths and depths. They observed a direct relationship between the alignment of human dermal fibroblasts and aspect ratios of the channel type patterns. While aspect ratios as small as 0.01 induced significant alignment (60%), 80% alignment was achieved with an aspect ratio of 0.05. The maximum aspect ratio required for 95% alignment was 0.16. This study indicates that within a certain range the aspect ratio can be used for controlling cell response to substrate topography without distinguishing the effects of width and depth. However, it is important to point out that when the grating surface is wider than cell width, the probability of lateral cell spreading is high. Thus, obtaining cell-type-specific contact guidance thresholds by the help of the abovementioned prediction theories can be useful to tailor the cellular microenvironment.

Cell alignment on physically patterned surfaces is a widely used strategy, in some cases together with chemical patterns. The effect of chemical patterns [29, 30] or synergistic effects of physical and chemical patterning [31–34] were also studied intensely. Generally, in order to control cell adhesion and alignment, molecules such as poly(-L-lysine) (PLL), peptides, fibronectin, laminin, collagen, bovine serum albumin, and SAM (self-assembled monolayers) are patterned by soft lithography techniques. In some cases, instead of synergistic effects of chemical and physical patterning, one of the cues can overcome the other one. For example, when Charest et al.  [33] used grooves (4 *μ*m depth, 8 *μ*m width) overlaid with an orthogonal chemical pattern (10 *μ*m adhesive lanes with spacing ranged from 10 to 100 *μ*m), physical topography determined the alignment of osteoblast-like cells. Another means to produce patterned surfaces is to use thermoresponsive polymers. These “smart” polymers, based, for example, on a poly(N-isopropylacrylamide) backbone with* n*-butyl methacrylate side chains, are capable of a reversible transition from hydrophilic to hydrophobic state when their temperature is lowered by a few degrees (around its low critical solution temperature of 32°C). Thermoresponsive polymers can be used for cell sheet engineering to treat a wide range of diseases, from corneal dysfunction to oesophageal cancer, tracheal resection, and cardiac failure as growth substrates for cells [35]. By using patterned thermoresponsive surfaces, it is possible to pattern cell sheets when the application requires cellular alignment.

Biological tissues are hierarchically organized from nanometer-to-centimeter scale. For instance, the average roughness of bone tissue is 32 nm and bone tissue has a hierarchical structure composed of collagen and hydroxyapatite [36]. During bone mineralization, the hydroxyapatite crystals form micro- and nanocomposites with collagen fibers. Thus, preparing biomimetic surfaces which present synergistic effects of micro- and nanostructures is expected to provide additional advantages. Two separate studies have examined the behavior of osteoblasts derived from bone marrow stromal cells on micropit and nanonodule hybrid topography of TiO_2_ [37, 38]. They created nanonodules with diameters of 100 nm, 300 nm, and 500 nm by self-assembly technique and demonstrated that 300 nm nanonodule containing substrates created the most promising environment for osteoblast differentiation and bone-titanium integration. These results are in agreement with more recent studies [39, 40], which have indicated that the presence of micron and submicron-scale surface roughness on the same surface can accelerate bone differentiation. In another study, surface features which are similar in scale to osteoclast resorption pits were used to study* in vitro* bone formation in basal medium [41] Here, the pit dimensions were as follows: depth: 330 nm, diameters: 20, 30, and 40 *μ*m, and centre-centre spacing: 50, 60, and 90 *μ*m. Osteopontin expression was relatively high in the human osteoblasts grown on the larger diameter (30 and 40 *μ*m) pits. In addition to expression of osteogenic markers, mature calcium depositions were shown by alizarin red staining on these substrates.

In the last decade, several studies reported that micro- and nanopatterned surfaces can be a valuable tool for directed growth [15, 42] and differentiation [19, 32, 43, 44] of neurites. In one of the recent studies, Migliorini et al.  [45] analyzed the effect of nanopillars on differentiation of embryonic stem cell derived progenitors in the absence of biochemical factors and observed an increase in the neuronal yield with increasing pillar height from 35 to 400 nm. Pan et al. [46] tested the effects of nanograting substrates with different widths on human induced pluripotent stem cells (hiPSCs). Gene expression profiling by real-time PCR and immunostaining showed significant upregulation of neuronal markers on nanostructured substrates either with solely topographical cues or combined with preneuronal induction. A width of 350 nm, in particular, induced the highest neuronal marker expression. The responsiveness of the cells to nanometer scale cues stems from their specific interactions with extracellular matrix (ECM), which is covered in the following section.

## 3. Microenvironmental Effects of Extracellular Matrix

The ECM comprises of a wide range of molecules including collagen, elastin, laminin, fibronectin, various glycoproteins, proteoglycans, and polysaccharides [47, 48]. Each ECM component provides different functionalities to this complex network made of structural proteins (e.g., collagen and elastin), adhesive proteins (e.g., fibronectin and vitronectin), and glycosaminoglycans (e.g., hyaluronic acid and heparin sulphate) by presenting different structural and biochemical properties [49]. The microenvironment created by ECM surrounding adherent cells is essential to their survival. ECM is a physical support to physiological cells; the signals for functional orientations such as migration, proliferation, and even survival are transduced from ECM. The absence of cell adhesion to ECM induces cell death by apoptosis [50]. This particular type of apoptosis is named* anoïkis* (Greek word which means “homelessness” or loss of home). This phenomenon was first described in epithelial cells [51] and contributes to maintain tissue homeostasis [52]. In physiological conditions, adherent cells are protected from* anoîkis* by the binding to ECM and the resulting activation of intracellular survival signalling pathways. The loss of* anoikis* induction signal constitutes a hallmark of cancerous cells and contributes to the formation of metastasis [53, 54]. Thus, presentation of an ECM mimic to the cells in tissue engineering applications is important. The three-dimensional organization of the ECM has a regulatory effect on cell cycle as seen in mammary epithelial cells as the ECM suppresses apoptosis, suggesting that ECM signaling is defined by the organization of the cells within a tissue, that is, cell shape, intercellular spacing, and 3D position. These factors determine cellular response to signals.

The microenvironment created by ECM components such as adhesive proteins or glycosaminoglycans maintains tissue stability and cell behavior. Bone matrix, for instance, contains 90% of collagen type I and only 5% of noncollagenous proteins like osteocalcin, osteonectin, fibronectin, or hyaluronan and mineral compounds which are essential to conserve osteoblasts phenotype [55], whereas culturing chondrocytes on type I collagen induces their dedifferentiation [56]. Furthermore ECM components selectively influence cell adhesion and shape as described by Schlie-Wolter et al. [57]. Hence, cell morphology directed by the interaction with ECM induces modifications of their behaviour and subsequently their fate [58].

One of the main examples of cell signaling is integrin-mediated signaling for cell adhesion where the connection requires structures of focal adhesion that contain complex mixture of proteins. Cell adhesion to ECM is led by transmembrane heterodimeric integrin receptors. During development, integrins facilitate tissue morphogenesis by determining which ECM components the cell would bind to. Integrins are the major mediators of cell-ECM contacts and they are essential to the outside-in transmission of signals from cell microenvironment [59]. Integrin and ligand bindings lead to the formation of focal adhesion complexes which are linked to the intracellular actin cytoskeleton [60, 61]. Another example of this structure-dependent ECM signaling pathway is in tyrosine kinases [62]. For cell binding and migration, integrin signaling modulates the cell signaling pathways of transmembrane protein kinases such as receptor tyrosine kinases (RTK). RTK are high-affinity cell surface receptors for many polypeptide growth factors, cytokines, and hormones. The study of receptor tyrosine kinase (RTK) signaling led to the understanding of how an extracellular signal is transmitted to the nucleus to induce a transcriptional response [63]. Other nonintegrin adhesion receptor families include selectins, cadherins, immunoglobulins, proteoglycans, and some other laminin-binding proteins. In short, this mode of interaction conveys biochemical and positional information by which the cell can know how and when it should undertake a particular activity.

ECM is coupled to cytoskeletal and signalling effector elements which direct crucial downstream functions, such as cell growth, survival, and transcriptional activity [64]. Biochemical and biomechanical modifications of ECM microenvironment are transmitted to cells and induce the resulting changes in their behaviour [65]. Cell mechanosensing is mediated by focal adhesion contacts [66]. Indeed, physical and mechanical forces in cell microenvironment lead to changes in cell morphology and differentiation. Not only the composition of the ECM has direct effects on cell behaviour, but also its physical properties. Stiffness of bone ECM is essential to maintain osteoblast phenotype whereas chondrocytes dedifferentiate where they are cultured on a rigid matrix [67]. Elasticity of ECM determines also the differentiation of progenitor cells [68]. Furthermore, physical modifications of an adhesive protein such as fibronectin are sufficient to influence cell activities. The stretching of fibronectin alters its binding to ligands [69] and more importantly fibronectin conformation regulates integrins attachment which controls downstream cell behaviour [70]. Another type of ECM component variation is biochemical. Glycosylation is one of the most abundant protein modifications having a role in protein stability, secretion, and function. The O-glycosylation in particular is essential in cell adhesion. Zhang and Hagen demonstrated that loss of glycosylation disrupts adhesion of epithelial cells and more generally influences cellular microenvironment [71]. Moreover, glycosylation of adhesive proteins like laminin or fibronectin also stimulates cancerous cell proliferation and dissemination [72].

All differentiated cells have a cell type specific protein expression profile with multifunctional criteria that is responsible for development and protein regulation. This protein expression creates an output signal to be used by cells to control their roles. Changes in the protein expression profile or mutations that result in down- or upregulation of specific proteins can be the causes of cardiac, muscular, or mental illnesses. Hyper- and hyposensitivity responses to the strength of stimuli can also cause sickness in the body. In order to model such illnesses, tissue engineering and biomaterials studies concentrated on producing artificial ECM networks that are made up of synthetic polypeptides or peptide-conjugated synthetic polymers that present bioactive ligands and respond to cell-secreted signals to enable proteolytic remodeling. The production of such biomaterials can be used in differentiating stem cells into neurons [73]. The goal of this approach is to mimic the properties of ECM. The areas of biomimicry are specific cell adhesion, degradation by proteolytic processes involved in cell migration and tissue remodeling, and the ability to control cellular functions such as ECM synthesis. An example of such systems is the use of photopolymerizable polyethylene glycol (PEG) based hydrogels as tissue engineering scaffolds. This material showed, when modified with necessary signals, that it can interact with cells in a manner similar to that of natural ECM, especially in transmitting bioactive signals that control tissue formation and cell phenotype.

ECM microenvironment is not permanent; changes during aging were observed in different organs with variable times of onset. Due to the specific interactions between different tissues, cells, and their surroundings, the cells modify their own environment by reshaping their ECM components into the correct configuration that allows the growth of the functioning tissues which have specific architecture and characteristics. ECM components are essential to stem cell maintenance and subsequently to support tissue regeneration [74].

## 4. Phenotype Control and Stem Cell Differentiation via Microenvironmental Cues

Tissue homeostasis requires a certain level of phenotypic plasticity from resident cells and also the involvement of circulating cells. The most apparent manifestation of this need is observed upon injury where the inflammatory reaction mediated by immune cells, such as neutrophils and macrophages, decides how an implant, transplant, or an engineered tissue integrates with the body. The phenotypes of the immune cells in the microenvironment have a significant effect on the final outcome. Moreover, many tissues depend on several different cell types with given phenotypes. The quality of bone tissue depends on the interaction between osteoblasts, osteoclasts, and osteocytes. Respiratory epithelium not only has a ciliary epithelium layer but also requires basal cells and glandular cell components.

**Figure 2:**
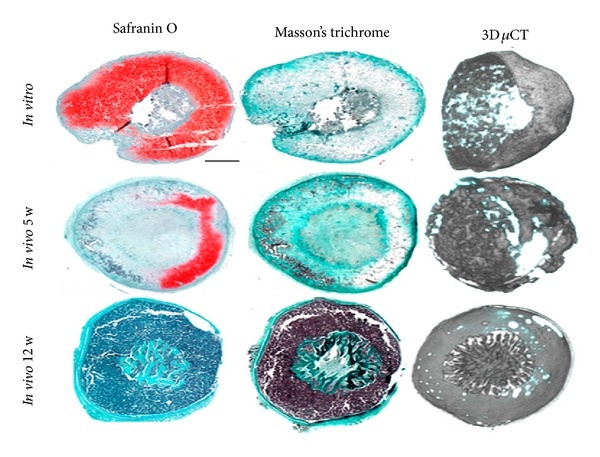
Bone formation via endochondral pathway. An* in vitro* formed artificial cartilage successfully forms a bone containing bone marrow within 12 weeks. The* in vitro* grown tissue is a cartilaginous one as evidenced by the extensive safranin O staining; over time, the cartilaginous tissue has been gradually replaced by bone tissue, as can be seen by the extensive Masson's Trichrome staining. Micro-CT images also showed the development of a bone like structure within 12 weeks. Reproduced from [8].

One of the new paradigms in tissue engineering is the utilization of developmental pathways for engineering tissues [75]. In one of the recent demonstrations of “developmental tissue engineering” [76], Scotti et al. were able to produce a bone organ with functioning bone marrow by putting the human mesenchymal stem cells through an endochondral bone formation route, that is, formation of bone organ via a cartilaginous tissue step [8]. This was achieved via production of hypertrophic cartilage tissue, by application of IL-1*β* and subsequent subcutaneous implantation. By applying both physical and chemical microenvironmental controls, they were able to push the initial hypertrophic cartilage structure to produce several cell types with their proper phenotypes, which demonstrates the strength of the developmental tissue engineering methods (Figure 2) [8].

The most active literature concerning cell phenotype in tissue engineering is the research on stem cells. Stem cells (SCs), a subset of cells with replenishing ability and the potential of differentiation into various types of mature cells, are categorized into two main groups, namely, embryonic stem cells (ESCs) and adult stem cells (ASCs). It has been shown that the intrinsic genetic programs within these cells and some extracellular regulatory signals control the ability of SCs to proliferate and differentiate into different functional mature cell types [77].

Stem cells reside in a specialized microenvironment called stem cell niche which provides the stem cells with extracellular cues to allow their survival and identity. This niche is a key regulator to the stem cell behavior because it ensures a quiescent and low metabolic environment to prevent exhaustion. It is believed that microenvironmental properties of the niche provide a good balance between the ability of SCs to renew themselves and the ability to differentiate into mature cells so that continuous tissue regeneration occurs. A major part of the cell niche is the ECM (extracellular matrix) which possesses the specific mechanical, biochemical, and biophysical properties for tissues and controls the overall cell behavior [78]. The composition of the ECM provides full support to the niche through its physical and structural properties. The main extrinsic signals that regulate stem cell behavior are those coming from ECM.

Given its three-dimensional organization, the ECM provides an environment that aids in the integration of the signals derived from the cell-ECM interactions in order to allow proper “maintenance of stem cell homeostasis” [78]. The cell-ECM interactions are basically triggered by receptors present on the cell membrane, like integrin as described before. However, studies found that the nonintegrin receptors are the ones that contribute the most to stem cells homing during transplantation. Novel techniques have been developed to observe the interaction between stem cells and ECM proteins and how this interaction influences their fate. Among the factors that influence stem cell fate are ECM adhesion, its stiffness, and its topography [79]. For example, the effect of micro-/nanotopography on stem cells has been recently demonstrated. Oh et al. [80] demonstrated that human MSCs can differentiate into osteoblasts under the influence of only nanotopography of culture substrates. Another example is micropatterned islands, created with specific shapes to observe cell behavior at single-cell level. The degree of spreading of human epidermal stem cells was observed by Connelly et al. [81]. The authors stated that when the shape of the island was changed from elongated to circular, epidermal stem cells showed an increase in their differentiation ability. On the other hand, human MSCs revealed a dependence on the area of the island, that is, while round cells favoured adipogenesis, whereas cell spreading resulted in osteogenesis [82].

**Figure 3:**
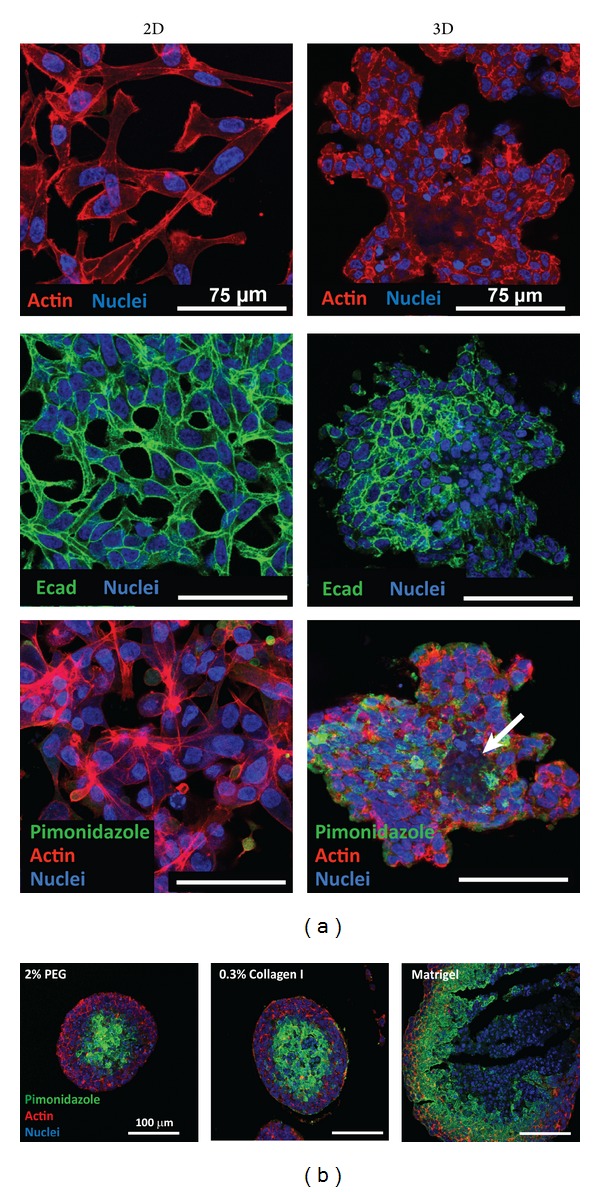
Manipulating the cell microenvironment in 3D via encapsulation within hydrogels. Encapsulation of prostate cancer cells within PEG hydrogels resulted in more pronounced cell-cell contacts as evidenced by E-cadherin staining (a) and also formation of a necrotic core within the cell aggregates as shown by pimonidazole staining (b). All scale bars are 75 *μ*m for (a) and 100 *μ*m for (b). Reproduced from [9].

In addition to topographical cues, soluble factors like growth factors and cytokines are very important in initiation and control of SC differentiation [83]. Tissue engineering has become an important stem cell application field with the aim of increasing the quality of life. Therefore, researchers have focused more on finding appropriate cues via utilization of biomaterials that could control the cellular environment and monitoring complex cellular levels. Both natural and synthetic materials based biomaterial scaffolds have served to understand the role of chemical cues in controlling stem cell behaviour. It is crucial to direct SCs to differentiate into the right cell type, at the right time and location; therefore, specific cues have been investigated in the* in vivo* microenvironment and have been studied in the* in vitro* systems that mimicked the natural conditions. Controlled microenvironments have been designed to direct stem cell differentiation into the desired mature cell type. Stem cell researchers emphasize the need of a 3D environment instead of 2D since differences have been observed in their self-renewal capacity, differentiation, adhesion, and migration ability. Cellular morphology has been shown to vary depending on the biomaterials structure (2D or 3D) and material type. Human mesenchymal stem cell shape was observed to be round when entrapped in 3D hyaluronic acid hydrogel [84] and elongated when seeded onto fibrous scaffolds or 2D biodegradable elastomer [85]. There are other effects regarding the encapsulation of cells as demonstrated by encapsulation of prostate cancer cells (LNCaP) in polyethylene glycol (PEG) hydrogels, which changed their cell-cell contact formation and response to androgen stimulation where these effects are also relevant to the differentiation of stem cells within confined environments [9] (Figure 3).

## 5. Cell Microenvironment Control via Delivery of Soluble Bioactive Agents

**Figure 4:**
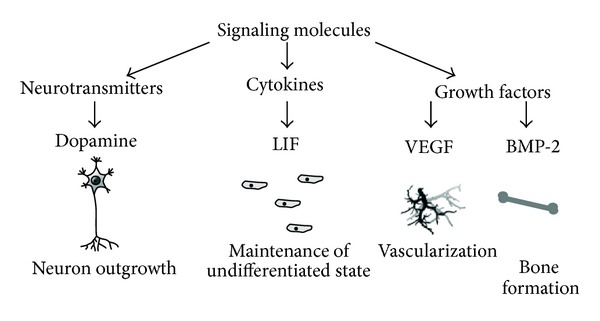
The main types of soluble factors that have distinct effects on the cellular behaviour at both single cell and tissue level. Controlled delivery of such factors and their regulated presence in cell microenvironment is an indispensable tool in tissue engineering research. Reproduced from [10].

Another way to control the cell microenvironment is via delivery of bioactive molecules such as drugs, hormones, or growth factors. Variation in the signaling microenvironment might cause perturbations in the signaling processes which are at the root of multiple pathologies, including cancers, diabetes, and many other diseases [63]. Growth factors can regulate activation, growth, proliferation, migration, and differentiation of cells which are crucial for events such as angiogenesis or osteogenesis [86]. Recent studies have focused on inserting signaling molecules such as growth factors and cytokines into biomaterials (Figure 4). Some examples of altering cell behaviour to such molecules are induced vascularisation (new blood vessel formation from fibroblast growth factor 2—FGF-2), regeneration of neurons (from nerve growth factor, NGF), retention of stem cell phenotype (from immobilization of cytokines to maleic anhydride copolymer thin-film coating), and providing an environment that helps cell survival and proliferation. Epidermal growth factor (EGF), incorporated with the matrix material, increases cell attachment to the implanted matrix and increases the spreading of mesenchymal stem cells [10].

However, these molecules have high instability and very short biological half time and can be enzymatically digested or deactivated while in physiological fluids [87, 88]. Besides, growth factors or drugs need to reach specific location to be effective; thus, their systemic introduction is not a viable way to control their concentration in specific target areas [89]. To overcome these limitations, it was necessary to develop delivery vehicles with growth factors or drugs incorporated within tissue engineering scaffolds. To illustrate this fact, it has been shown that bolus injection of growth factors such as VEGF is less effective than a sustained and localized delivery via biodegradable hydrogels to achieve blood vessel formation [90]. In the case of bolus injection, VEGF was not localized in the target area and stayed only for 72 h, whereas with delivery from biodegradable alginate hydrogel 95% of the growth factor was at the ischemic site (improvement of biodistribution), and it stayed at that location for more than 15 days and its bioactivity was higher (possibly due to the protection from denaturation).

One way to achieve control over local bioactive molecule concentrations is the immobilization of growth or differentiation factors. Mainly, the ECM harbors a lot of growth factor binding proteins. This localization of growth factors by the ECM and their signaling contributes to the establishment of a gradient for the soluble, diffusible morphogens, which play vital roles in shaping the developmental processes [91]. The binding of growth factors to the ECM is regulated by the GAG side chains. One important application of this is the regulation of specific gene expression which is done by using growth factor-ECM interaction, that is, by controlling the growth factor presence via their interaction with ECM [92].

Incorporation of bioactive factors into scaffold can be achieved by two different ways, mainly through covalent and noncovalent immobilization [93–95]. The first approach is based on the covalent binding of the molecule to the scaffold via chemical reaction such as immobilization of VEGF using* N*-(3-dimethylaminopropyl)-*N*′-ethylcarbodiimide hydrochloride chemistry (EDC) [96]. The noncovalent approach is based on interaction of the molecule with the polymer matrix such as electrostatic interaction, hydrogen bonding, or physical entrapment. In the case of incorporation via electrostatic interaction, charged material is required such as polyelectrolytes or gelatin hydrogels. In all these different approaches, the release will be triggered by scaffold degradation, diffusion of the molecule through the material, or cleavage (enzymatic or hydrolysis) of the covalent bond between the molecule and the scaffold material. For example, with the addition of bioactive motifs (from bone morphogenetic protein 2 or osteopontin), it was shown that osteoblast adhesion and the responsiveness to the protein were dependent on the cell adhesive motif from osteopontin. The cell interaction with the protein demonstrated* in vitro* bone formation in a month [97].

These delivery systems were developed with different kind of materials. Two main categories can be identified: (i) natural materials such as collagen, alginate, gelatin, and poly-L-lysine; (ii) synthetic materials such as PLLA, PEG, and PCL [95]. To be an efficient carrier system, these materials must fulfill some requirements: (i) biocompatibility, (ii) biodegradability, and (iii) release of active factors in a controlled spatiotemporal way [98]. Release of growth factors from scaffolds is mainly governed by two mechanisms: (i) diffusion through the material and (ii) degradation rate of the material [86, 99]. Release profile of bioactive molecules is a key parameter to control cell microenvironment. Depending on the application, such as enhancement of angiogenesis, stem cell differentiation, or disease treatment, bioactive factors need to be released for specific time points at specific rates. In the case of degradable scaffolds, the release of molecules can be tuned by varying the degradation profile of the material or the molecule diffusion. The degradation rate of the scaffold can be changed by crosslinking to reinforce the structure and delay the release. Gelatin is a biomaterial obtained by denaturing collagen. It is a good ECM mimicking material for cells [100]. Moreover, gelatin is biodegradable and has been used for a long time in medical field. Gelatin is also a very useful material for drug incorporation because it can be positively (basic gelatin) or negatively charged (acidic gelatin) depending on collagen processing method (acid or alkaline process) so it can complex both positively or negatively charged molecules [101, 102]. This provides a level of versatility which is not available with other commonly used biomaterials. Gelatin hydrogels are systemically cross-linked with different agents such as genipin, transglutaminase, or EDC/NHS because, unless cross-linked, the structure of the physically gelled gelatin hydrogels or films is too weak and the degradation is too fast [103–105]. For other materials, different techniques are available. For example, to control degradation rate of alginate hydrogels, a partial oxidation of the polymer chains rendered the hydrogel degradable by hydrolysis [90]. This strategy has been used to create a delivery vehicle for VEGF. Synthetic hydrogels can also be used to encapsulate and release bioactive molecules. Hyperbranched polyester hydrogels capable of encapsulating hydrophobic molecules such as growth factors or specific drugs (e.g., dexamethasone) have been developed. These hydrogels are photocross-linkable via incorporation of methacrylate group. Normally, it is very difficult to entrap hydrophobic agents in hydrogels. In this case, a sustained release of 8 days was achieved mainly through hydrolysis of ester backbone [106].

Polyelectrolyte multilayer structures (L-b-L, layer by layer) are also used to design delivery systems because L-b-L films are easy to produce; they can act as a reservoir for bioactive molecules [107] and their properties such as permeability, thickness, and charge density can be easily changed and they can be easily coated on implants [108]. The only problem with these films for drug delivery application is the fast release of molecule due to the mobility of polyelectrolyte chains within the film. To solve this problem, recently, a double entrapment system has been developed for VEGF to achieve a long term release [109]. This strategy was based on the twofold control over the release by VEGF containing PCL nanoparticles loaded in polyelectrolyte multilayer film. The mechanism of the release was the following: either PCL nanoparticles containing VEGF were hydrolyzed and then VEGF diffused in the LBL film and then out; or the particle will diffuse out of the film and then hydrolyzed. With this system, a sustained release of 7 days was achieved [109]. To prevent the fast release of drug out of LBL film, another system was developed by adding a mechanosensitive cap as a barrier on top of LBL reservoir films. A bioactive agent was loaded in PLL/HA film and a PAH/PSS barrier was built on top of it. Barrier is cracked under stretch which enables the diffusion of an enzyme (trypsin) within the reservoir and the PLL/HA is enzymatically degraded leading to the release of drug [110]. Layer by layer technique (LBL) with polyelectrolytes can also be used in particle form (i.e., particles formed by polyelectrolyte multilayers) [111]. Using this technique, a stimuli-responsive controlled drug release has been developed in order to release bioactive agents. This system was based on the absorption of the agent on mesoporous silica sphere and then the deposition of a multilayer capping barrier PAH/PSS. The release of the encapsulated molecule was further triggered by change of pH (pH = 1.4) or by change of ionic strength through NaCl concentration of the release media (10 mM NaCl). At higher pH value or lower ionic strength, the PAH/PSS layer acted as a capping barrier since it does not allow bioactive agent diffusion and that explains why this system is appropriate for a controlled and sustained release of bioactive molecules [112].

In some other applications, the delivery of multiple bioactive factors with different release kinetics is required. In tissue engineering, for example, angiogenesis and osteogenesis are regulated by the action of multiple growth factors and all of them need to be released in a specific temporal way. Richardson et al. have investigated the dual delivery of VEGF and PDGF, two growth factors necessary for blood vessels formation. PLG particle with lyophilized VEGF and PLG microspheres containing encapsulated PDGF were used [113]. All these particles were mixed together and a porous PLG scaffold was made using high pressure carbon dioxide fabrication process. These growth factors release profiles were not the same: 1.7 pmol/day for VEGF for the first seven days mainly due to VEGF diffusion out of the scaffold and from 0.10 to 4.7 pmol/day for PDGF depending on degradation of polymer particle using different formulations [113]. In the field of regenerative periodontal therapy, an interconnected macroporous Dex-GMA (glycidyl methacrylate dextran)/gelatin hydrogel scaffold was developed for the dual delivery of two different growth factors: BMP-2 and IGF-1. These growth factors were encapsulated in Dex-GMA/gelatin microparticles; basic gelatin (negatively charged) was used to encapsulate BMP-2 and acidic gelatin (positively charged) for IGF-1 encapsulation. As Dex-GMA is enzymatically degradable but not hydrolytically degradable, other Dex-GMA microparticles were prepared with neutral gelatin to encapsulate dextranase in order to further trigger the release of growth factors by enzymatic degradation of the Dex-GMA microparticles. All these particles were mixed together and cross-linked via irradiation in order to make the interconnected macroporous scaffold. This mechanism of delivery based on the degradation of microparticle followed by degradation of the scaffold enables the sustained release of growth factors for a period of 20 days [114]. Some disease like periodontitis required the multiple deliveries of antibacterial, anti-inflammatory, antiresorptive, and osteogenic agents. These molecules must be delivered in a very specific order to be effective. To fulfill this requirement, a multilayered device was designed. This laminate structure is made by the association of cellulose acetate phthalate (CAP) and Pluronic F-127(P) and these polymers can be further eroded. The different drugs have been incorporated in a specific order in every stratum by mixing them with the polymer solution and every layer is separated by one or two CAPP blank layers in order to slow the erosion of the structure and delay the release of molecules. This system was able to perform the release of four different drugs in a specific temporal sequence just by unidirectional erosion of the structure for more than 120 hours depending on the condition used [115]. The other important cell microenvironment parameter that needs to be mimicked is the changes induced on cells via dynamic stimuli, such as mechanical stimuli. The spatial and temporal variations of cell microenvironment play vital roles in various paracrine and endocrine cell signaling, 3D tissue remodeling, alteration in stem cell niches cancer progression, and migration of various cells. For these reasons, the dynamic nature of the cell microenvironment is vitally important as discussed below.

## 6. Dynamic Aspects of Cell Microenvironment

The native ECM microenvironments of cells are highly heterogeneous in three-dimensional space [116] and they go through continuous and dynamic remodeling with time [117]. The interactions of the ECM components with cells are of reciprocal nature, that is, while cells constantly produce, break down, rearrange, and realign components of ECM to change its properties, and any change in ECM in turn regulates the activities and the behavior of the cells [47]. The cell-ECM interactions are highly dynamic and complex; hence, a detailed understanding of the dynamic aspects of cellular microenvironment in terms of spatial and temporal variations of different chemical, mechanical, and biological stimuli is highly important in tissue or organ engineering.

Bioreactors are a crucial part of tissue engineering research as they are the main means to exert mechanical stimuli to the cells in tissue engineering constructs. Bioreactors are systems which enable the continuous replacement of nutrients and gases either by constant agitation (as in the case of spinner flasks) or via perfusion. Most of the bioreactor systems have several entry ports which enables the introduction of different bioactive agents in a controlled manner. Moreover, many bioreactors have the capacity to directly apply physiologically relevant mechanical stress/strain conditions (such as tensile, compressive shear stress according to the target tissue). They mimic the mechanical microenvironment, for functional engineered tissues that undergo mechanical loading (articular cartilage, tendons, heart valves, etc.) under* in vivo* conditions. They are designed to increase the efficiency of exchange of metabolites, oxygen, nutrients, and waste removal within the cell microenvironment and as a result to enhance cellular penetration in 3D scaffolds and to have a better and rapid cellular expansion [118]. For this aim, there exist perfusion bioreactors for culturing encapsulated stem cells and those for cell cultivation on 3D scaffolds.

Mimicking the dynamic mechanical environment is also considered in designing functional bone implants in conjunction with micro-/nanotopographical features. These studies pointed out that osteoblasts change their morphology, gene expression, and matrix mineralization by either introducing surface topography on biomaterials or mechanical stimulation [119, 120]. Prodanov et al. [121] tested the simultaneous effects of nanotextured surface (300 nm wide and 60 nm deep grooves) and mechanical stretching in terms of cell attachment, ECM formation, and osteoblast differentiation. It was shown that by dual stimulation (nanogrooved surface and 8% of strain) the expression of fibronectin and Cfba synergistically increased 2-fold in comparison to nanotextured surface alone. Such combined effects of topography and mechanical stimulation were also observed by other groups [41, 122–124].

Mechanical stimulus by cyclic stretching, fluid flow [121], and hydrostatic compressive pressure has also been used to align cells. In several studies, cardiac, ligament, and tendon derived fibroblasts, myoblasts, vascular smooth muscle cells, and osteoblasts were subjected to mechanical forces under* in vivo* conditions. Regardless of cell types, cells cultured on a 2D substrate that is subjected to uniaxial cyclic stretch tend to align perpendicular to the direction of principal cyclic strain [122, 125, 126]. There are several studies that have used mechanical stimuli together with micro/nanopatterning for cell alignment. It has been shown that when cells grown whether on patterned surfaces with micron sized, or nanosized groove/ridge patterns or on unpatterned [127] substrates subjected to cyclic stretching orient themselves in the direction perpendicular to the applied strain. It should be noted that applied strains in these examples were parallel to the direction of the groove axis. However, there are studies showing that tendon fibroblasts and osteoblasts aligned along the direction of micro grooves regardless of the stretching direction, which suggests that the topographical cues at this scale might be a more important determinant for the cell alignment direction [122, 128]. For instance, mechanical loading, topographical patterning, and surface chemical treatment can also be combined to engineer cell alignment* in vitro*. The combined effects of cyclic strain and substrate microtopography on the alignment of bovine vascular SMCs have been investigated by Ahmed et al. [124], where they observed that the organisation of actin fibers was dominated by cyclic strain application and the shape of cell nuclei was controlled by the patterns.

### 6.1. Dynamic Control of Cell Microenvironment Using Microfluidics

Researchers have designed cell-laden matrices in 3D space to mimic functions of human tissues and organs* in vitro*. Many of these structures also change over time (4D biology) [117]. Pioneering work by Petersen et al. revealed that mammary epithelial cells formed a normal acinus structure when encapsulated in a 3D material but aberrantly displayed cancerous phenotypes when cultured on a 2D substrate [129]. Other examples [130, 131] revealed that the materials based presentation and timed removal of the peptide RGDs can enhance differentiation of mesenchymal stem cells into chondrocytes. Thus, the spatial and temporal control of microenvironment has been implemented in various studies. The synergistic effects of chemical factor gradients, cell-cell-interactions, mechanical sensing, and coordinated cell movements in tissue formation can be achieved through various microscale and microfluidic technologies. Microfluidic devices offer novel platforms for precise control and variation of cellular microenvironments in dynamic, automated, and reproducible ways. The use of microfluidic systems in controlling the cellular microenvironment offers numerous advantages, such as the following: (i) they have the potential to simulate real tissue microenvironments including multiple cell types and ECM proteins into a 3D structure, (ii) they use a very small number of cells and small quantities of reagents, typically in the nanoliter to microliter range, (iii) they allow precise control over cell density and cell shape as well as environmental cues such as attachment matrices containing self-assembling proteins and gel based substances, (iv) they provide the ability to precisely control the mechanical properties (e.g., elasticity, rigidity, and strain), chemical properties (e.g., ligand density and orientation), and topographic properties (e.g., patterning of surfaces with substances having different cell-substrate affinity), and (v) they allow high throughput analysis and complete automation of the processes. Due to these advantages, recently microfluidic devices have been widely used in controlling the cell/tissue microenvironment in tissue engineering applications. The variation of the local mechanical properties [132], chemical properties, and topographic features [133, 134] has been achieved using microfluidic platforms. The control over localized ECM [135–138], chemical gradient [139, 140], and fluid flow [141, 142] has also been achieved.

The applications of microfluidic technologies in tissue engineering and biomedical engineering, in general, have become widespread, such as for development of blood vessels and 3D vascularized tissues [143] and use of microfluidic platforms in controlling the cell microenvironment for gene therapies [144].

Gilmore et al. [145] used an affinity capture technique in a microfluidic chamber for capturing and maintaining rotavirus double-layered particles (DLPs) in a liquid environment. In another study, Walker et al. [146] used a laminar flow and diffusion mediated, gradient based microfluidic device to infect the cells at many different concentrations of virus simultaneously within a single microfluidic channel. The laminar flow and diffusion have been used for establishing gradient in many other studies as well [147–150].

Xu et al. [151] used a three-layer microfluidic device for* in situ* monitoring of the infection process of cells by a recombinant virus in real time. They also performed drug screening assays on the microfluidic chip with a tree-like concentration gradient.

Na et al. [152] used soft lithography based technique to create cell adherent and repellent areas on a substrate, thereby depositing cells in desired micropatterns and forming plaques of controlled size, shape, and cells number. Microfluidic platforms have also been used as bioreactors [153] containing separate compartments for production, preservation, and transduction of viruses or compounds on a single microfluidic device. Thus, microfluidic systems and microscale technologies present novel platforms for controlling cell microenvironment for various cell and tissue engineering applications.

## 7. Microenvironment Monitoring

The level of control over microenvironment is directly related to our level of understanding the mechanisms underlying the dynamic processes. One of the challenges in tissue engineering is continuous monitoring of cellular activities within 3D, generally opaque, thick structures. There are several exciting technologies that have been developed for visualisation of 3D structures that are currently being applied to tissue engineered scaffold.

For screening purposes of biomaterials microenvironment on cells, microelectromechanical systems (MEMS) have been utilized. Features at length scales from 1 *μ*m to 1 cm can be controlled with this technique for stem cell analysis [154]. Response of stem cells toward different microenvironmental signals has been studied by using robotic spotters, which can test cell-matrix interactions with a very high throughput [155, 156]. Another possibility to monitor the cell behaviour at process level is the real-time imaging of cell microenvironment in microfluidic chambers [135].

For direct real-time monitoring of the processes within engineered tissues, one proposed method is the incorporation of biosensors within the artificial tissues. This is a direct extension of implantable biosensors for clinical applications, which can be generalized under continuous monitoring of metabolites such as glucose [157]. Currently, such systems are nearly available and only hindered by the long-term problems of foreign body reaction and biofouling which impede their reliability and precision [158]. In addition to these problems, a remodellable tissue engineering scaffold provides a complex microenvironment which also has degradation byproducts of the scaffold material, host cells, implanted cells, and their secretions. Recently, a three-parameter* in vivo* biosensor system was proposed by Kubon et al. [159] which can simultaneously measure oxygen, pH, and electrical impedance to access the reaction to a given biomaterial. Such a system would provide the necessary information concerning oxygenation levels, infection, and level of integration for a given volume of the engineered tissue microenvironment. Although this system has not been used* in vivo* yet, it has been validated in an* ex vivo* chorioallantoic membrane assay (CAM assay) system [160].

Noninvasive visualisation techniques are another way to monitor cell microenvironment. Techniques such as optical coherence tomography [161] or nonlinear microscopy techniques [162, 163] can provide relevant information about the scaffold microenvironment and its interaction with the cells. For assessment of the implanted cell activity within the host, modified signal producing cells can be utilised. By using firefly luciferase (ffLuc-MSC) expressing MSCs, Kidd et al. [164] were able to monitor the dispersion of the cells* in vivo* and found out that the MSCs show a preference to accumulate if a tumor or an inflammation site is present in the host mice. This tropism is related to the presence of a cytokine microenvironment which is more permissive and chemoattractive for their incorporation, which provides a guideline to understand how to control the interaction of the host tissue with the implanted engineered tissue. Aside from cellular localization, another crucial information for thick engineered tissues is the level of oxygenation, particularly within the depth of the structure, where the lack of nutrients and oxygen can lead to necrosis. A method to obtain relevant information about the cell microenvironment is to incorporate stimuli-responsive structures that would signal the relevant changes in the microenvironment. Acosta et al. [165] developed a fluorescent microparticle based oxygen sensing system that enables the monitoring of hypoxia and hyperoxia conditions within the 3D tissue engineering scaffolds. A similar method with phosphorescent nanoparticles was used to detect oxygen levels* in vivo* [166].

## 8. Future Directions

Despite the significant progress made during the last decade, designing materials to control cellular microenvironment remains an important goal. Also, challenges remain in dynamically controlling the cell microenvironments temporally and spatially. Toward modulation of dynamics, the use of stimulus-sensitive linkers, protecting groups, and exposing mechanisms may provide paths forward. It may be possible to exploit biomechanical and biochemical stimuli to expose cryptic biomolecular signals in synthetic biomaterials, as also occurs in some natural ECM molecules [73]. Microarray based material development has received great attention. Materiomics, which allows high throughput testing of complex material surfaces for specific applications, provides the necessary information for producing more complex cell microenvironments [167]. It allows researchers to place a large collection of materials onto two-dimensional substrates in a spatially numbered matrix. This way, the effects of several different properties of materials on cells have been studied simultaneously. The arrays are in the form of combinatorial polymer microarrays [156, 168, 169], peptide microarray [170], combinatorial ECM protein microarrays [171–174], and topographical microarrays  [175–178]. These approaches could dramatically increase and accelerate discovery of next generation biomaterials. Moreover, for regenerative medicine and tissue engineering applications, understanding the behaviours of cells in 3D is going to move the field forward. Immunomodulation via modulation of macrophage phenotype or via design of biomaterials, bottom-up techniques for production of multifunctional, multicellular structures, real time biosensing and linked bioactive agent delivery systems within the engineered scaffolds will improve the control of biomedical engineers on artificial tissues further.
